# Eating disorders as core business for mental health clinicians working in a public mental health service

**DOI:** 10.1186/2050-2974-1-S1-O60

**Published:** 2013-11-14

**Authors:** Rachel Barbara-May, Paul Denborough

**Affiliations:** 1 Alfred Child & Youth Mental Health Service, Australia

## 

This presentation will demonstrate how public mental health systems can deliver accessible, effective and client-centred eating disorder treatment. It will provide an overview of service development over time aimed at increasing workforce capacity, responsiveness of service provision, improved access and better integration of all essential elements of care. The presentations will describe and discuss this service delivery initiative in the Southern region of Melbourne, looking at barriers, enablers and implications for policy and planning. An overview of the development of an integrated mental health and paediatric service for young people with eating disorders at Alfred Health Child and Youth Mental Health Service will be provided. Data on service provision and outcomes will be discussed, along with a case example illustrating a typical case presenting for treatment at this service.

This abstract was presented in the **Children and Youth Treatment and Service Development** stream of the 2013 ANZAED Conference.

